# Antiviral Properties of the Natural Polyphenols Delphinidin and Epigallocatechin Gallate against the Flaviviruses West Nile Virus, Zika Virus, and Dengue Virus

**DOI:** 10.3389/fmicb.2017.01314

**Published:** 2017-07-11

**Authors:** Ángela Vázquez-Calvo, Nereida Jiménez de Oya, Miguel A. Martín-Acebes, Emilia Garcia-Moruno, Juan-Carlos Saiz

**Affiliations:** ^1^Departamento de Biotecnología, Instituto Nacional de Investigación y Tecnología Agraria y Alimentaria Madrid, Spain; ^2^Centro di Ricerca Viticoltura Enologia (CREA-VE), Consiglio per la Ricerca in Agricoltura e l’Analisi dell’Economia Agraria Asti, Italy

**Keywords:** *Flavivirus*, polyphenol, delphinidin, epigallocatechin gallate, virucidal, West Nile virus, Dengue virus, Zika virus

## Abstract

The *Flavivirus* genus contains important pathogens, such as West Nile virus (WNV), Zika virus (ZIKV), and Dengue virus (DENV), which are enveloped plus-strand RNA viruses transmitted by mosquitoes and constitute a worrisome threat to global human and animal health. Currently no licensed drugs against them are available, being, thus, still necessary the search for effective antiviral molecules. In this line, a novel antiviral approach (economical, simple to use, and environmental friendly) is the use of natural compounds. Consequently, we have tested the antiviral potential of different polyphenols present in plants and natural products, such as wine and tea, against WNV, ZIKV, and DENV. So that, we assayed the effect of a panel of structurally related polyphenols [delphinidin (D), cyanidin (Cy), catechin (C), epicatechin (EC), epigallocatechin (EGC), and epigallocatechin gallate (EGCG)] on WNV infection, and found that D and EGCG inhibited more effectively the virus production. Further analysis with both compounds indicated that they mainly affected the attachment and entry steps of the virus life cycle. Moreover, D and EGCG showed a direct effect on WNV particles exerting a virucidal effect. We showed a similar inhibition of viral production of these compounds on WNV variants that differed on acidic pH requirements for viral fusion, indicating that their antiviral activity against WNV is produced by a virucidal effect rather than by an inhibition of pH-dependent viral fusion. Both polyphenols also reduced the infectivity of ZIKV and DENV. Therefore, D and EGCG impair the infectivity in cell culture of these three medically relevant flaviviruses.

## Introduction

The *Flavivirus* genus contains important pathogens such as Zika virus (ZIKV), Dengue virus (DENV), yellow fever virus (YFV), Japanese encephalitis virus (JEV), and West Nile virus (WNV), among others. As flaviviruses, WNV, ZIKV, and DENV are enveloped plus-strand RNA viruses transmitted by mosquitoes and constitute a worrisome threat to global human and animal health (Martin-Acebes and Saiz, 2012; Saiz et al., 2016; Pang et al., 2017). WNV is maintained in an enzootic transmission cycle between mosquitoes and birds, but it can also infect other vertebrates, such as horses and humans (Martin-Acebes and Saiz, 2012). Whereas most WNV infections in humans are asymptomatic, ∼20% of cases develop a febrile illness that can progress to severe neurological disease, including meningitis, encephalitis, and flaccid paralysis, which can be fatal (Sejvar, 2014). DENV is the most common mosquito-transmitted infective agent, responsible of over 300 million unapparent cases per year in humans, of which near 100 million are clinically relevant and cause dozens of hundreds deaths yearly (Pang et al., 2017). On the other hand, since the first detection of ZIKV in the Americas in 2014, the virus has explosively spread thru the continent resulting in the infection of millions of people (Saiz et al., 2016). Until then, ZIKV infection was characterized as causing a mild disease with exceptional reports of an association with Guillain-Barré syndrome, (GBS; Blazquez and Saiz, 2016; Saiz et al., 2016). However, during the recent outbreaks in America, a 20 times increase in the number of cases of microcephaly in fetuses and newborns, and a surprising number of GBS cases have been linked to ZIKV infection (Blazquez and Saiz, 2016). In any case, and despite the efforts of the scientific community, currently no drug against any of these viruses has been licensed, and, thus, the search for effective antiviral compounds is a current milestone in the field (Martin-Acebes and Saiz, 2012; David and Abraham, 2016; Saiz et al., 2016; Saiz and Martin-Acebes, 2017).

In this regard, a novel antiviral approach (economical, simple to use, and environmental friendly) is the use of natural compounds (Martinez et al., 2015), such as the polyphenols present in plants and natural products, such as wine and tea (Hsu, 2015). Polyphenols constitute important metabolites of plants and are generally involved in defense against ultraviolet radiation or aggression by pathogens. These compounds have antioxidant properties and present protection against development of cancers, diabetes, osteoporosis, and cardiovascular and neurodegenerative diseases (Pandey and Rizvi, 2009; Li et al., 2014). Polyphenols have also demonstrated potential antibacterial, antifungal, and antiviral activities. For instances, the antiviral effect of epigallocatechin gallate (EGCG), the most abundant polyphenol in green tea, has been already reported for several viruses: hepatitis C virus, chikungunya virus, hepatitis B virus, herpes simplex virus type 1 virus, influenza A virus, vaccinia virus, adenovirus, reovirus, vesicular stomatitis virus, and, very recently, for ZIKV (Ciesek et al., 2011; Calland et al., 2012, 2015; Huang et al., 2014; Weber et al., 2015; Carneiro et al., 2016). Likewise, other polyphenols have also been described to present antiviral activity, such as honokiol, baicalein, naringin, and quercetin (Zandi et al., 2011, 2012; Johari et al., 2012; Fang et al., 2015).

In this report, we have analyzed the effect of different polyphenols on WNV infection and found that of EGCG and delphinidin (D) inhibited the multiplication of these flaviviruses in cell culture. Even more, both polyphenols also showed a direct effect on WNV, ZIKV, and DENV particles, more probably due to a virucidal activity.

## Materials and Methods

### Reagents

Delphinidin chloride (D), cyanidin chloride (Cy), catechin (C), epicatechin (EC), epigallocatechin (EGC), and EGCG (Extrasynthèse) were resuspended in dimethyl sulfoxide (DMSO) and used at a final concentration of 10 μM.

### Cells, Viruses, Infections, and Virus Titrations

Vero cells (ATCC CCL-81) were cultured as described (Martin-Acebes et al., 2014; Merino-Ramos et al., 2014). All infectious virus manipulations were performed in our biosafety level 3 (BSL-3) facilities. Viral strains used were: WNV-NY99, WNV mutants with increased resistance (Martin-Acebes et al., 2013) or sensitivity (Martin-Acebes and Saiz, 2011) to NH_4_Cl, ZIKV PA259459, and DENV-2. Unless otherwise specified, cells were incubated with the corresponding virus for 1 h at 37°C, then viral inoculum was removed, and incubations were continued in culture medium containing 2% fetal bovine serum [time that was considered as 1 h post-infection (p.i)]. Viral titres were determined by plaque assay in semisolid agarose medium at 24 h p.i (Martin-Acebes and Saiz, 2011). The multiplicity of infection (MOI) used in each experiment was expressed as plaque forming units (PFU)/cell, and is indicated in the corresponding figure legend.

### Cell Viability

The lack of toxicity of the compounds was evaluated by determining the cellular ATP content with a CellTiter-Glo luminescent cell viability assay (Promega).

### Quantitative RT-PCR

West Nile virus RNA was extracted from the supernatants of infected cultures using Speedtools RNA virus extraction kit (Biotools B&M Labs S.A.), whereas cell-associated WNV RNA was extracted using TRI Reagent (Thermo Fisher Scientific). The amount of viral RNA copies was determined by quantitative RT-PCR (Blazquez et al., 2016). Genomic equivalents to PFU per milliliter were calculated by comparison with 10-fold serial dilutions of viral RNA extracted from previously titrated sample (Blazquez and Saiz, 2010).

### LysoSensor Assay

Fluorescent labeling of acidic organelles was performed using LysoSensor Green DND-189 probe (Life Technologies). The LysoSensor reagent exhibits a pH-dependent increase in fluorescence intensity upon acidification. Vero cells were seeded on glass bottom 35-mm dishes (Ibidi), washed with PBS, and treated (4 h) with 25 mM NH_4_Cl, or 10 μM of either D or EGCG in EMEM supplemented with 25 mM HEPES pH 7.4. Then, the culture medium was replaced by fresh medium containing 1 μM of LysoSensor probe, and cells were incubated during 5 min at 37°C, washed three times with the same media without LysoSensor probe, and observed under a fluorescence microscope. Images were acquired and processed using the same microscope settings. Control cells were treated in parallel with the same amount of drug solvent (H_2_O for NH_4_Cl and DMSO for polyphenols).

### Data Analysis

Analysis of variance (ANOVA) was performed with the SPSS (v.15) statistical package (SPSS Inc.). Data are presented as the mean ± standard deviation (SD). Differences with *P*-values of <0.05 were considered statistically significant.

## Results

### Delphinidin and Epigallocatechin Gallate Inhibit WNV Infection

**FIGURE 1:**
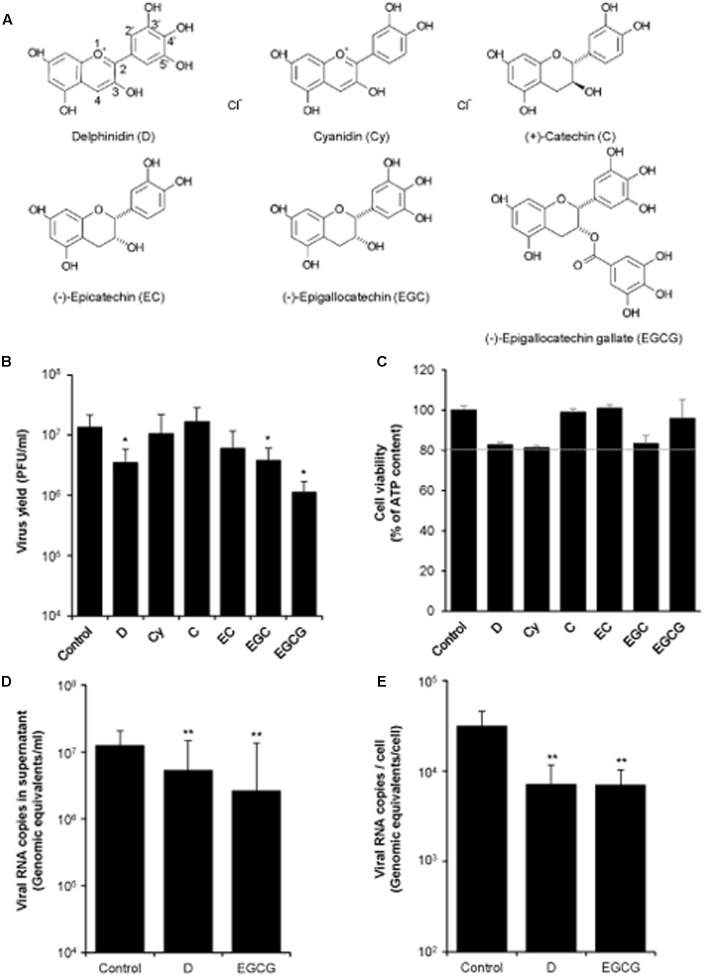
Effect of different polyphenols on WNV infection. **(A)** Schematic representation of the different polyphenols tested. **(B)** Vero cells were infected with WNV NY99 (MOI of 0.5 PFU/cell) and the different polyphenols (10 μM) were added after the first hour of infection (adsorption time). Virus yield in culture supernatant was determined by plaque assay at 24 h p.i. **(C)** Mock-infected Vero cells were treated (24 h) in parallel with 10 μM of each polyphenol and the cell viability was determined by measuring the cellular ATP content. Dashed line indicates 80% of cell viability of control cells. **(D)** Quantification by qRT-PCR of genome-containing particles in culture supernatant of Vero cells infected and treated as in **(B)**. **(E)** Amount of cell-associated viral RNA in cell cultures infected and treated as in **(B)** determined by qRT-PCR at 24 h p.i. Data are represented as mean + SD. Statistically significant differences are indicates: ^∗^*P* < 0.05; ^∗∗^*P* < 0.005.

The effect of a panel of structurally related polyphenols (**Figure 1A**) on WNV infection was analyzed in cell culture. To this end, Vero cells were infected with WNV and treated with the different polyphenols. Among the compounds tested, only D, EGC, and EGCG significantly reduced WNV yield at 10 μM concentration (**Figure 1B**). At this concentration no remarkable toxicity was observed, as cell viability was higher than 80% of the control (**Figure 1C**). Since D and EGCG exerted the highest inhibition against WNV infection, they were further analyzed. Thereby, quantitative RT-PCR showed that both polyphenols significantly reduced the amount of viral RNA in the supernatant of infected cultures (**Figure 1D**), which is compatible with the observed reduction on the release of infectious particles (**Figure 1B**), as well as the amount of cell-associated viral RNA (**Figure 1E**).

### Delphinidin and Epigallocatechin Gallate Act Directly on the Viral Particle

**FIGURE 2:**
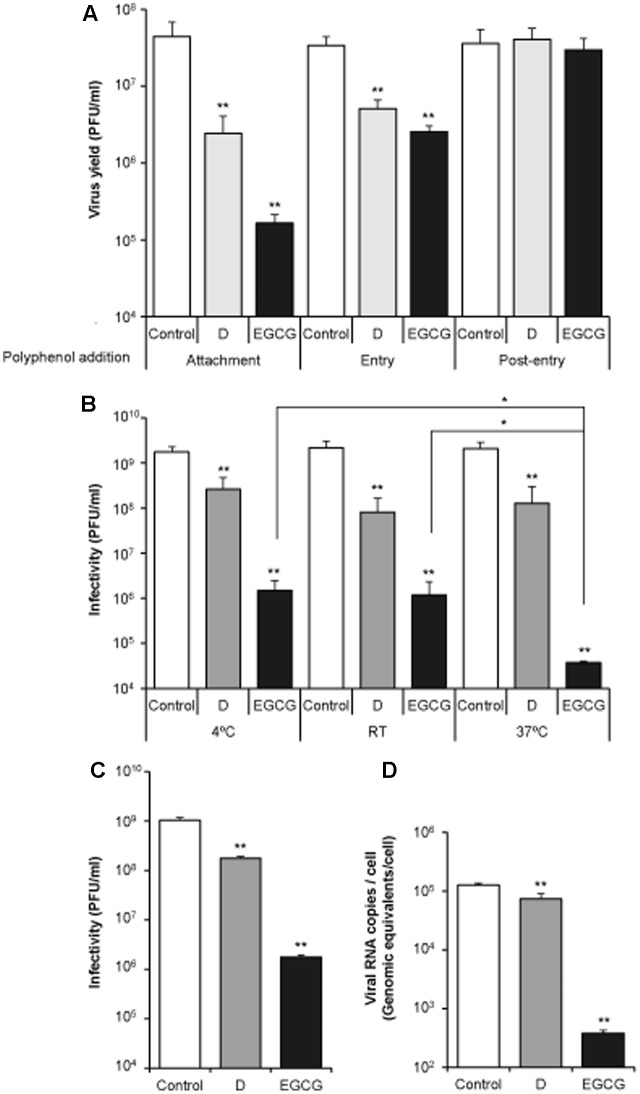
Delphinidin (D) and epigallocatechin gallate (EGCG) inhibit WNV infection by exerting a virucidal effect that impairs an early step of the infection. **(A)** DC and EGCG only inhibit WNV when added at an early step of infection. Vero cells were incubated with WNV (MOI of 1 PFU/cell) at 4°C to allow viral attachment and, then, shifted to 37°C to promote virus entry and infection. The polyphenols were added during this attachment step (Attachment), during the two first hours at 37°C (Entry), or from 2 h after infection until the end of the experiment (Post-entry). Virus yield in supernatant was determined by plaque assay at 24 h p.i. **(B)** Virucidal effect of the polyphenols on WNV infection. WNV (6 × 10^8^ PFU) were incubated (1 h) with 10 μM of each polyphenol, or the same volume of drug vehicle (DMSO) as a control, at 4°C, RT, or 37°C. Then, the infectivity in each sample was determined by plaque assay. **(C,D)** Viral infectivity and amount of cell-associated viral RNA in cell cultures infected with pretreated virus as in **(B)** (37°C) assayed by plaque assay and qRT-PCR at 24 h p.i, respectively. Data are represented as mean + SD. Statistically significant differences are indicates: ^∗^*P* < 0.05; ^∗∗^*P* < 0.005.

In order to dissect the step of the infection affected by the polyphenols, D and EGCG were added at different times of infection (**Figure 2A**). To analyze if they interfere with (i) viral attachment: cells were incubated with the polyphenols together with the virus for 1 h at 4°C, thus allowing virus binding to the cell monolayers, but preventing viral entry (**Figure 2A**, Attachment). (ii) Viral entry: viruses were adsorbed (1 h) to cell monolayers at 4°C to allow virus attachment and synchronize viral entry. Then, viral inoculum was removed and fresh medium (warmed at 37°C) containing the polyphenol was added for 2 h, time at which the medium was replaced by fresh medium without the drugs. So that, polyphenols were only present during the first 2 h of infection (**Figure 2A**, Entry). (iii) Late infection steps: cell monolayers were infected as in (ii), incubated with fresh medium warmed at 37°C for 2 h, and then the medium was replaced by fresh medium containing the polyphenols throughout the rest of the experiment (**Figure 2A**, Post-entry). Using this experimental approach it was noticed that both D and EGCG exhibited antiviral effect only at early steps of the viral infection.

To further explore whether D and EGCG inhibit viral infection by acting directly on the viral particle, WNV (6 × 10^8^ PFU) was incubated with 10 μM of each compound during 1 h, and the remaining infectivity in each sample was determined by plaque assay (**Figure 2B**). Considering that flaviviruses can undergo temperature dependent conformational changes (Zhang et al., 2015), the assay was performed at different temperatures (4°C, RT, or 37°C). Incubation with D or EGCG reduced significantly the infectivity of WNV in all cases (**Figure 2B**). Even more, whereas no difference was observed among the temperatures assayed for D, in the case of EGCG incubation at 37°C resulted in a significantly higher reduction of WNV infectivity. Additionally, when viral inoculum was pretreated with the drugs for 1 h at 37°C prior to infect cell monolayers, a significant reduction on virus yield and the accumulation of intracellular viral RNA was noticed for D and EGCG in comparison to control samples (**Figures 2C,D**). These data further supported the virucidal effect of the compounds. Inhibitions, although relatively similar, were dose-dependent in a 1–10 μM range (data not shown). Overall, these results indicate that D and EGCG affect the early steps of WNV infection, most likely, by exerting a virucidal effect.

### The Antiviral Effect of Delphinidin and Epigallocatechin Gallate Is not Related to an Inhibition of Endosomal Acidification

**FIGURE 3:**
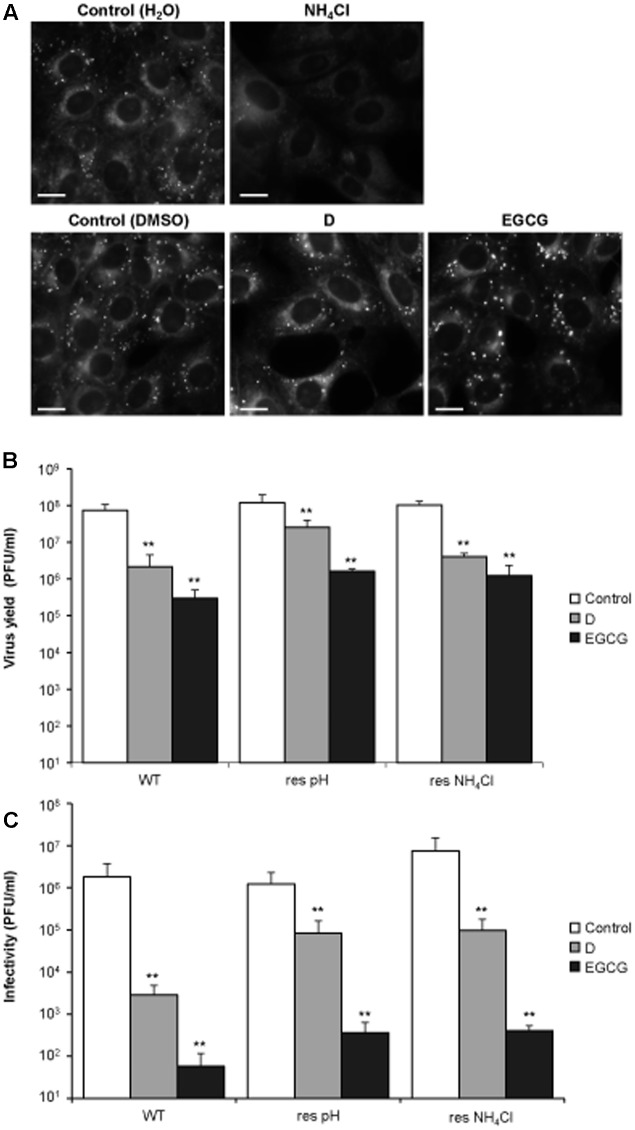
The antiviral effect of D and EGCG is not related to an impairment of endosome acidification. **(A)** DC and EGCG do not inhibit endosome acidification. Vero cells treated (4 h) with 25 mM NH_4_Cl, 10 μM DC, or 10 μM EGCG were incubated with LysoSensor probe during 5 min and analyzed by fluorescence microscopy. Bars: 10 μm. **(B)** Vero cells were infected (MOI of 1 PFU/cell) with WNV NY99 (termed WT), a mutant resistant to acidic-induced inactivation (termed res pH), or a mutant resistant to NH_4_Cl (termed res NH_4_Cl). DC and EGCG were added at 10 μM 1 h p.i and keep during the whole virus infection. The virus yield in the supernatants was determined by plaque assay at 24 h p.i. **(C)** 1–2 × 10^8^ PFU of the different WNV strains were incubated with 10 μM during 1 h at 37°C. Then, the remaining infectivity in each sample was determined by plaque assay. Data are represented as mean + SD. Statistically significant differences are indicates: ^∗∗^*P* < 0.005.

Polyphenols, such as catechins, can either inhibit (Imanishi et al., 2002) or enhance (Zhong et al., 2015) endosomal acidification. Therefore, and considering that the acidic pH within the endosomal system promotes viral fusion during WNV entry (Martin-Acebes and Saiz, 2012), we explored whether the antiviral effect of D and EGCG on WNV infection was related with a potential interference of these polyphenols with endosomal pH. To do so, cells were treated with the polyphenols and incubated with LysoSensor probe, which exhibits a pH-dependent increase in fluorescence intensity upon acidification (**Figure 3A**). As cells treated with NH_4_Cl, which blocks endosomal acidification, have been shown to inhibit WNV infection (Martin-Acebes et al., 2013) they were included in these experiments. Whereas control untreated cells stained with LysoSensor exhibited a fluorescence punctuated pattern that corresponded to acidic organelles, NH_4_Cl abolished the presence of these structures, thus confirming that this compound blocked the acidification of the endolysosomal system. Conversely, control cells were indistinguishable from D or EGCG treated-cells, suggesting that these compounds did not impair endosomal acidification (**Figure 3A**). Remarkably, both polyphenols also inhibited the infection of two WNV variants (**Figure 3B**) that differed on acidic pH requirements for vial fusion (Martin-Acebes and Saiz, 2011; Martin-Acebes et al., 2013). Even more, they also displayed virucidal effect against these mutant viruses (**Figure 3C**). Taken together, these results indicate that D and EGCG do not impair endosomal acidification, and suggest that their antiviral activity against WNV is produced, at least partially, by a virucidal effect rather than by an inhibition of pH-dependent viral fusion.

### Virucidal Effect of Delphinidin and Epigallocatechin Gallate against ZIKV and DENV

**FIGURE 4:**
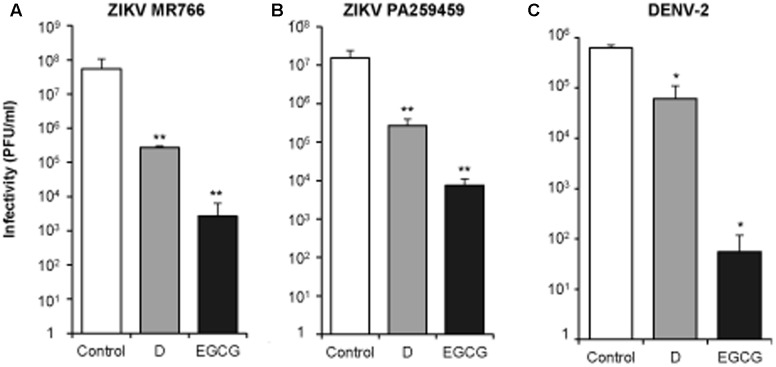
Delphinidin and EGCG exhibit virucidal effect against ZIKV and DENV. ∼10^6^ PFU of ZIKV MR766 **(A)**, ZIKV PA259459 **(B)**, or DENV-2 **(C)** were incubated (1 h at 37°C) with 10 μM DC or EGCG, or the same volume of drug vehicle (DMSO) as a control. Then, the remaining infectivity in each sample was determined by plaque assay. Data are represented as mean + SD. Statistically significant differences are indicates: ^∗^*P* < 0.05; ^∗∗^*P* < 0.005.

To extend the virucidal effect of D and EGCG to other medically relevant flaviviruses, ∼10^6^ PFU of two ZIKV strains of different genetic lineages, the African MR766 and the American PA259459 (**Figures 4A,B**), and of DENV-2 (**Figure 4C**) were incubated with 10 μM of D, or EGCG, for 1 h at 37°C, temperature at which WNV exerted the highest virucidal effect (**Figure 2B**). Results showed that both polyphenols reduced the infectivity of ZIKV and DENV, confirming that they exhibit a virucidal effect against medically relevant flaviviruses. In the case of ZIKV, the effect of D and, particularly of EGCG, was higher for the African strain (MR766) than for the American strain (PA259459).

## Discussion

Polyphenolic compounds from plant extracts possess many beneficial properties; including antiviral activities (Hsu, 2015; Parker et al., 2016; Yang et al., 2016). In this study, we have explored the *in vitro* antiviral capacity of different polyphenols, and shown that EGC, EGCG, and D inhibit WNV infection, and that the last two also inhibit DENV and ZIKV. Even more, in agreement with previous reports for viruses of different families (Ciesek et al., 2011; Calland et al., 2012, 2015; Huang et al., 2014; Weber et al., 2015; Carneiro et al., 2016), the antiviral activity of these compounds was only observed when the polyphenols were added during the early steps of the infection. Further analyses revealed that both, D and EGCG, act as virucidal agents by exhibiting a direct effect on WNV particles. The antiviral activity of D has been previously observed for HCV, a member of the *Flavivirida*e family (Calland et al., 2015), but this is the first study describing its antiviral activity against three medically relevant flaviviruses (WNV, ZIKV, and DENV).

The mode of action of EGCG and D is not yet well understood, but it has been hypothesized that, in the case of HCV, they might interact with the function of proteins at multiple binding sites (Calland et al., 2015). In this sense, molecular docking and dynamics simulation studies predicted a flavonoid binding pocket on the surface of the E protein of DENV, and showed that EGCG, baicalein, and quercetin potentially form interactions with this viral protein (Ismail and Jusoh, 2016). Therefore, and although further studies are needed it, it seems reasonable to assume a similar mechanism of action for the effect observed here. Notably, differences were observed in the inhibition exerted by EGCG and D to the two different ZIKV strains tested, one African (MR766) and one American (PA259459), especially by EGCG. This differences could be due to the different amino acid composition of the E protein, as the African strain exhibited a 4 amino acid deletion corresponding to the E protein 154 glycosylation motif found in most ZIKV strains and in many flaviviruses (Kuno and Chang, 2007; Baronti et al., 2014; Berthet et al., 2014). In fact, these two ZIKV strains have been recently reported to present different antigenicity and immunogenicity *in vivo* (Vazquez-Calv et al., unpublished). Moreover, EGCG showed higher virucidal activity against WNV at 37°C than at 4°C, probably because temperature-induced conformational changes (Zhang et al., 2015) allow more interactions between this compound and the WNV particle.

On the other hand, from a chemical point of view, the presence of a trihydroxyphenyl group at R2 in D and EGCG has been related to their anti-HCV activity (Calland et al., 2015). Accordingly, our results showed that an anti-WNV effect was only observed with those polyphenols having a hydroxyl group at R5’ (D, EGC, and EGCG), but not with those lacking it (Cy, C, and EC).

In addition to direct binding to the viral particle as an antiviral mechanism of action, it has been reported that these polyphenolic compounds may also interfere with the endocytic pathway by reducing virus uptake by endocytosis (Huang et al., 2014), or by modulating the endosomal pH (Imanishi et al., 2002; Zhong et al., 2015), which could impair pH-dependent viral fusion. Consistent with these observations, our results provide evidence that the tested polyphenols only have an antiviral effect when added at the early steps of WNV infection. However, no differences on endosomal acidification upon polyphenol treatment were observed. Furthermore, they also impaired the infection of WNV mutants differing in pH requirements for uncoating (Martin-Acebes and Saiz, 2011; Martin-Acebes et al., 2013). Therefore, all these data support that, in the case of WNV, their mechanism of action is not related to an inhibition of the pH necessary for viral fusion but, more likely, throughout a direct effect on the viral particle. In fact, other polyphenols have shown similar mechanisms of action, such as baicalein that exhibited anti-adsorption and virucidal activities against DENV and JEV (Johari et al., 2012), and naringin that presented anti-adsorption and anti-attachment activity against DENV (Zandi et al., 2012).

Due to their properties, polyphenolic compounds have been proposed as broad spectrum antiviral candidates (Colpitts and Schang, 2014; Hsu, 2015). Here, our results unveil the antiviral activity of two different polyphenols, D, and EGCG, against three clinically relevant members of the *Flavivirus* genus (WNV, ZIKV, and DENV), and point to them as potential scaffolds for future design of antiflaviviral compounds.

## Author Contributions

Conceived and designed the experiments: AV-C, NJ, MM-A, and J-CS. Performed the experiments: AV-C and NJ. Analyzed the data: AV-C, MM-A, EG-M, and J-CS. Contributed to the writing if the manuscript: AV-C, MM-A, NJ, EM-G, and J-CS.

## Conflict of Interest Statement

The authors declare that the research was conducted in the absence of any commercial or financial relationships that could be construed as a potential conflict of interest.
